# Development of a Highly Sensitive Analytical System for Measuring 17β-Estradiol Using Fluorescent Molecular Probes

**DOI:** 10.3390/s26092836

**Published:** 2026-05-01

**Authors:** Yoshio Suzuki

**Affiliations:** Health and Medical Research Institute, National Institute of Advanced Industrial Science and Technology (AIST), 1-1-1 Higashi, Tsukuba 305-8566, Ibaraki, Japan; suzuki-yoshio@aist.go.jp

**Keywords:** fluorescence, estrogen, aptamer, molecular probes, portable device

## Abstract

Easier measurement of 17β-estradiol could promote the early diagnosis and treatment of medical conditions in women. In this study, we developed a fluorescence-based assay using a nucleic acid aptamer labeled with a fluorescent dye for the detection of estrogen. Upon binding to 17β-estradiol, the aptamer undergoes a conformational change, resulting in a measurable change in fluorescence intensity. The assay enables rapid detection within 30 min, with a limit of detection of 0.2 pg/mL and a linear dynamic range of 1–1000 pg/mL. High selectivity toward 17β-estradiol was confirmed against structurally related compounds. The method was successfully applied to human saliva samples, demonstrating high sensitivity, precision, and reproducibility with recoveries of 98.8% and coefficients of variation below 3.0%. In addition, a compact desktop fluorescence detector was developed, allowing direct measurement in polymerase chain reaction tubes without sample transfer, thereby simplifying the procedure and minimizing sample loss. These results demonstrate that the proposed system provides a simple and practical platform for estrogen detection in biological samples and has potential applications in clinical and research settings.

## 1. Introduction

Estrogens, such as 17β-estradiol, are female steroid hormones produced in the ovaries and the placenta during pregnancy [1]. Estrogens act on the uterus to promote endometrial proliferation and prepare the uterus for pregnancy. They also play a critical role in organ formation and functions, such as sexual differentiation, bone growth, and brain development and maturation [2,3,4]. Furthermore, estrogens help maintain vascular health and regulate cholesterol levels to prevent arteriosclerosis. They are involved in the development of premenstrual syndrome (PMS), osteoporosis after menopause, arteriosclerosis, cerebral infarction, and high blood pressure [5,6]. Estrogen is also involved in protecting brain function, and its decline may increase the risk of developing Alzheimer’s disease [7,8]. Being able to easily measure these female hormones could facilitate the development of strategies to alleviate PMS symptoms, as well as promote early diagnosis and treatment of medical conditions in postmenopausal women. To achieve easier measurement, an analytical reagent that reacts selectively and rapidly with estrogen is required.

Methods for measuring 17β-estradiol in samples in vitro include high-performance liquid chromatography (HPLC), enzyme-linked immunosorbent assay (ELISA), and colorimetric analysis using gold nanoparticle aggregation [9,10,11,12,13,14]. The HPLC method involves separating a sample containing estrogen with HPLC, and then detecting the estrogen using an ultraviolet (UV) detector. This method has the disadvantage of being cumbersome and requiring specialized skills. The ELISA method is an immunological technique using antibodies that specifically bind to target substances. While it is possible to perform highly selective and sensitive analysis, repeated binding reactions between the target substance and the antibody are required; hence, measurements are time-consuming, often extending overnight or longer. This prevents rapid assessments. The colorimetric analysis using gold nanoparticle aggregation relies on the following phenomenon: when estrogen is added to a complex formed between gold nanoparticles and a nucleic acid that interacts with estrogen, the nucleic acid dissociates from the gold nanoparticle surface, forming a complex with the nucleic acid, simultaneously causing a color change due to the aggregation reaction of the gold nanoparticles. Although this measurement method is simple, challenges include low detection sensitivity. Therefore, it is essential to develop efficient and rapid methods that can be used to selectively determine and continuously detect changes in estrogen levels in organisms.

Aptamers are single-stranded nucleic acids selected through the systematic evolution of ligands by exponential enrichment (SELEX) process, enabling high-affinity and specific binding to target molecules [15,16], and fluorescent biosensors, including aptamer-based systems, have been extensively studied [17,18,19]. Estrogen-binding aptamers have been previously reported, with demonstrated binding affinity and selectivity toward 17β-estradiol [20,21]. Moreover, while homogeneous and rapid detection methods have also been explored [22], many still suffer from limited selectivity, insufficient sensitivity, or lack of applicability to biological samples without pretreatment [20,21,22].

When designing a molecular probe for detecting estradiol, it is important that the probe exhibits low fluorescence intensity before complex formation with estrogen and shows a significant increase in fluorescence upon complex formation. It is also necessary to incorporate a fluorescent substance into the molecular probe without compromising the efficacy of the estrogen recognition site. These considerations promote reduced background fluorescence, high sensitivity, high selectivity, and reduced influence of interfering substances.

**Figure 1:**
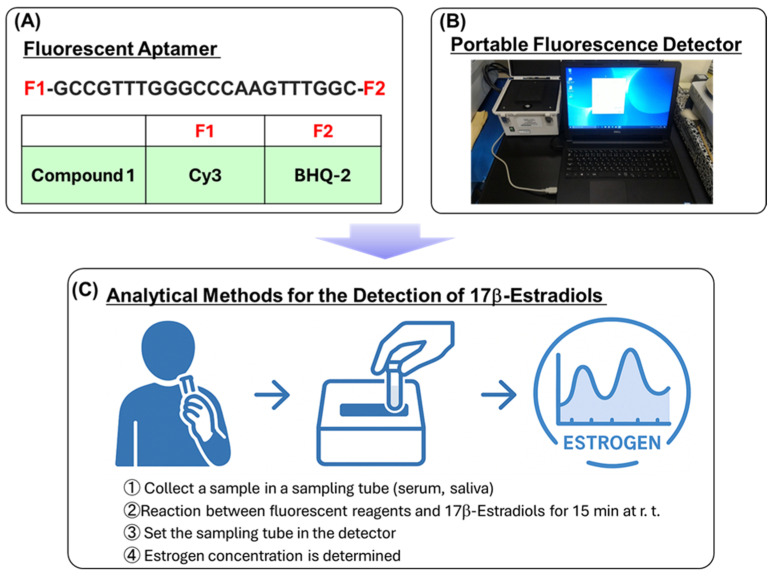
Chemical structure of the fluorescent aptamer compound **1** (**A**), external appearance of the portable fluorescent device (**B**), and research strategy for the detection of 17β-estradiol (**C**). In (**A**), the sequence of nucleic acids is shown in the upper section, while the fluorophore and quencher are shown in the lower section.

The aim of this study was to develop a fluorescent analytical reagent for detecting estrogen. The reagent is composed of a nucleic acid aptamer as the recognition site of estrogen, fluorophore (Cy3), and fluorescence quencher (BHQ-2). The aptamer sequence was designed based on existing evidence [13] to ensure that the structure of the aptamer changes before and after estrogen recognition, resulting in a large change in fluorescence intensity. The chemical structure of the fluorescent probe is shown in Figure 1A as compound **1**.

Moreover, a portable fluorescence detector was developed. The external appearance of the device is shown in Figure 1B. A typical fluorometer uses a 1 cm square cell or microplate, and the sample solution must be transferred into these containers during measurement. In this new analytical device, the prepared research sample can be measured in a polymerase chain reaction (PCR) tube; hence, it is not necessary to transfer the container before and after the measurement. This reduces the complexity of operation and sample loss. Additionally, the device is small and does not take up installation space. The experimental results clearly indicate that the fluorescent sensor developed in this study has excellent chemical characteristics for the detection of estrogen. Our research strategy is illustrated in Figure 1C. It is believed that the use of such a fluorescent analytical reagent will enable estrogen analysis to be performed easily, with high selectivity and sensitivity.

## 2. Materials and Methods

### 2.1. General Information

All chemicals used were of analytical grade and were purchased from Tokyo Chemical Industry (TCI, Tokyo, Japan), Wako Pure Chemical Industries, Ltd. (Osaka, Japan), Sigma–Aldrich (St. Louis, MO, USA), and GE Healthcare (Chicago, IL, USA). The synthesis of fluorescent aptamers was outsourced to GenScript Biotech Corporation (Nanjing, China).

Absorption spectra were recorded at 25 °C using a V-670 UV/visible spectrophotometer (JASCO, Tokyo, Japan), and fluorescence spectra were recorded at 25 °C using a FP-6500 fluorophotometer (JASCO, Tokyo, Japan).

A control serum, purchased from Wako Pure Chemical Industries, Ltd. (Osaka, Japan), contained immunoglobulin (Ig) G (1005 ± 100 mg/dL), IgA (193 ± 19 mg/dL), IgM (77 ± 7 mg/dL), complement component 3 (110 ± 11 mg/dL), complement component 4 (19.4 ± 1.9 mg/dL), C-reactive protein (0.79± 0.07 mg/dL), rheumatoid factor (23 ± 3 IU/mL), and antistreptolysin O (90 ± 13 IU/mL).

### 2.2. Compact Fluorescence Detector

**Figure 2:**
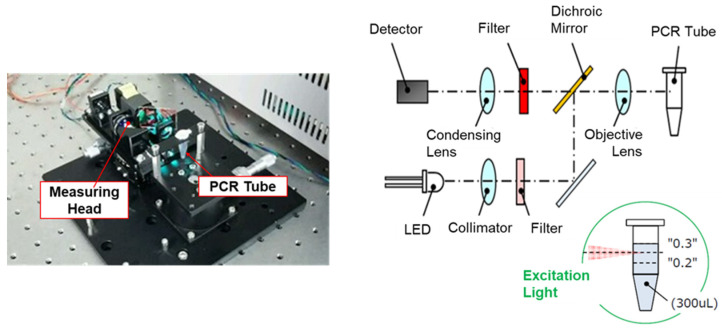
External appearance of excitation and fluorescence detection units in the portable fluorescent device (**left**), and the schematic diagram of optical layout (**right**). PCR: polymerase chain reaction.

The fluorescence detector was fully conceived and designed by the authors. Pulstec Industrial Co., Ltd. (Hamamatsu, Japan) was engaged solely as a contract manufacturer to fabricate the prototype device in accordance with the authors’ specifications. The experimental apparatus and its optical layout are shown in Figure 2. This apparatus was constructed using a plastic tube containing the sample, detection component, and data analysis component, which were controlled by a laptop. The detection unit was sealed from light, and a green light-emitting diode (LED; 531 ± 20 nm, Power: 24cd) was used as the excitation light source. The detector element was Photo Diode-IC. The excitation light filter transmission wavelength was 531 ± 20 nm, and the detection light filter transmission wavelength was 573 ± 20 nm. The excitation section is composed of an optical system including an objective lens, which focuses the light emitted from the LED light source and irradiates it onto a plastic tube containing the sample to mitigate potential optical interference or light scattering from the plastic PCR tube walls. The detection section is composed of an optical system including an objective lens; this section focuses the fluorescence induced by the excitation light onto a detector, which converts it to an electrical signal. The excitation/detection section is also configured coaxially using a dichroic mirror. The output voltage of the PD-IC is proportional to the intensity of the fluorescence. This device can measure the fluorescence intensity of a specific wavelength and its change over time.

### 2.3. Measurement of Compound ***1*** After Reaction with 17β-Estradiol

All measurements were performed in triplicate (n = 3). Compound **1** was dissolved in HEPES buffer solution (20.0 mM, pH 7.2) to obtain a concentration of 2.0 nM. After mixing 150 μL of compound **1** solution with 150 μL of 17β-estradiol, solutions of foreign substances in HEPES buffer solution (20.0 mM, pH 7.2), or human body fluids such as serum and saliva, the reaction mixture was incubated for 30 min, and fluorescence intensities were recorded. The final concentration of compound **1** was 1.0 nM.

### 2.4. Storage Stability of Compound ***1***

The lifetime of the fluorescent reagent was measured by incubating compound **1** at 4 °C. The residual activities were monitored by investigating the fluorescence intensity after reaction of compound **1** with 17β-estradiol at each time point.

## 3. Results and Discussion

**Figure 3:**
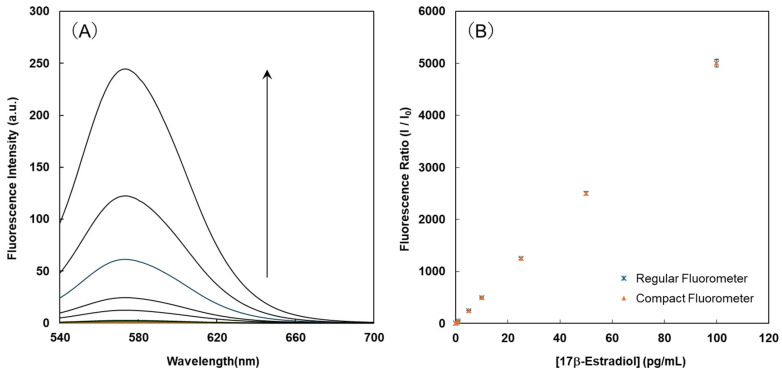
Changes in the fluorescence spectra of compound **1** before and after adding 17β-estradiol (**A**). A comparison of 17β-estradiol concentration fluorescence intensities observed with the conventional fluorescence spectrophotometer with those observed with the portable fluorescence detector (**B**). [1] = 1.0 nM; [17β-estradiol] = 0~100 pg/mL; excitation wavelength = 530 nm for the fluorescence spectrophotometer, and 531 ± 20 nm (LED) for the portable fluorescence detector. The y-axis indicates the fluorescence ratio (I/I_0_), where I is the fluorescence intensity of compound **1** at 569 nm before and after adding 17β-estradiol, and I_0_ is the fluorescence intensity of compound **1** at 569 nm. LED: light-emitting diode.

The fluorescence spectra of compound **1** were monitored at 25 °C using a spectrofluorometer (FP-6500 fluorophotometer, JASCO, Tokyo, Japan.) before and after adding various concentrations of 17β-estradiol. Experiments were conducted with compound **1** at 1.0 nM and 17β-estradiol at 0–100 pg/mL in HEPES buffer (20.0 mM, pH 7.2) for 15 min, which provides a near-physiological environment suitable for maintaining the aptamer structure and stable fluorescence of Cy3. The incubation time was set to 15 min, at which point the fluorescence signal reached a plateau. These results are shown in Figure 3A. Compound **1** exhibited a very low emission rate, whereas the 1·17β-estradiol complex exhibited notable increases in fluorescence intensities and strong green emissions. The fluorescence intensity of compound **1** at 570 nm increased from 0.04 to approximately 242.3 following the reaction with 17β-estradiol, corresponding to a 6000-fold increase.

The observed fluorescence change shown in Figure 3A is likely due to modulation of the fluorescence resonance energy transfer (FRET) efficiency between Cy3 and BHQ-2 upon estradiol binding, which may alter the distance and/or orientation between the fluorophore and quencher. Similar mechanisms have been reported in FRET-based aptamer sensors [23,24].

The emission intensities of compound **1** at 569 nm were plotted as a function of the 17β-estradiol concentration. Figure 3B shows the resulting typical calibration graph of the dependence of the intensities on the 17β-estradiol concentration under optimum experimental conditions. This plot exhibits a linear relationship between the emission intensities of compound **1** and the fluorescence intensity. The detection limit was 0.2 pg/mL for 17β-estradiol (signal-to-noise ratio of 3.0).

In the assessment of the fluorescence intensity of compound **1** when the estradiol concentrations were changed, the results obtained with a regular fluorometer were compared with those obtained with a compact fluorescence detector. The resulting data are shown in Figure 3B. The slope of the calibration curve obtained with the regular fluorometer was the same as that obtained with the compact fluorescence detector. The compact fluorescence detector was able to measure low concentration ranges of estrogen adequately. These results confirmed the reliability of the values obtained with the developed compact fluorescence detector.

**Figure 4:**
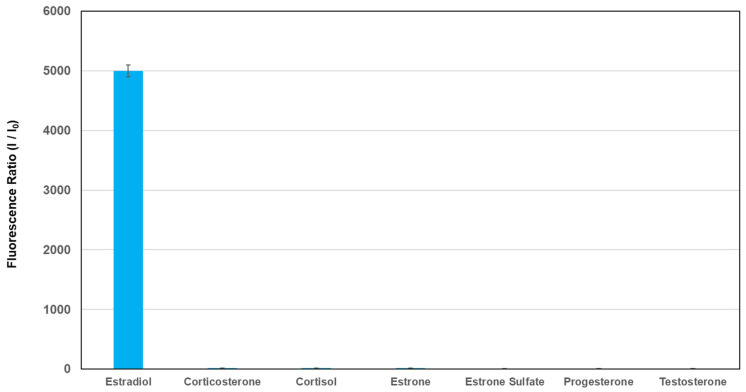
Fluorescence intensity of compound **1** at 569 nm before and after adding 17β-estradiol and various foreign substances. [1] = 1.0 nM; [Additives] = 100 pg/mL; excitation wavelength = 531 ± 20 nm. The y-axis indicates the fluorescence ratio (I/I_0_), where I is the fluorescence intensity of compound **1** at 569 nm before and after adding 17β-estradiol, and I_0_ is the fluorescence intensity of compound **1** at 569 nm.

To confirm the selectivity of compound **1** for 17β-estradiol, the fluorescence ratio of compound **1** at 569 nm was monitored before and after adding various foreign substances, such as corticosterone, cortisol, estrone, estrone sulfate, progesterone, and testosterone. The concentrations of 17β-estradiol and other compounds were all 100 pg/mL. The results are shown in Figure 4. A large increase in the fluorescence intensity of compound **1** occurred when a solution of 17β-estradiol was added. Conversely, the fluorescence ratios of compound **1** after adding various interfering compounds were much lower than those of 17β-estradiol, and large changes in fluorescence intensities were not observed. This result suggested that compound **1** had high selectivity for 17β-estradiol.

**Figure 5:**
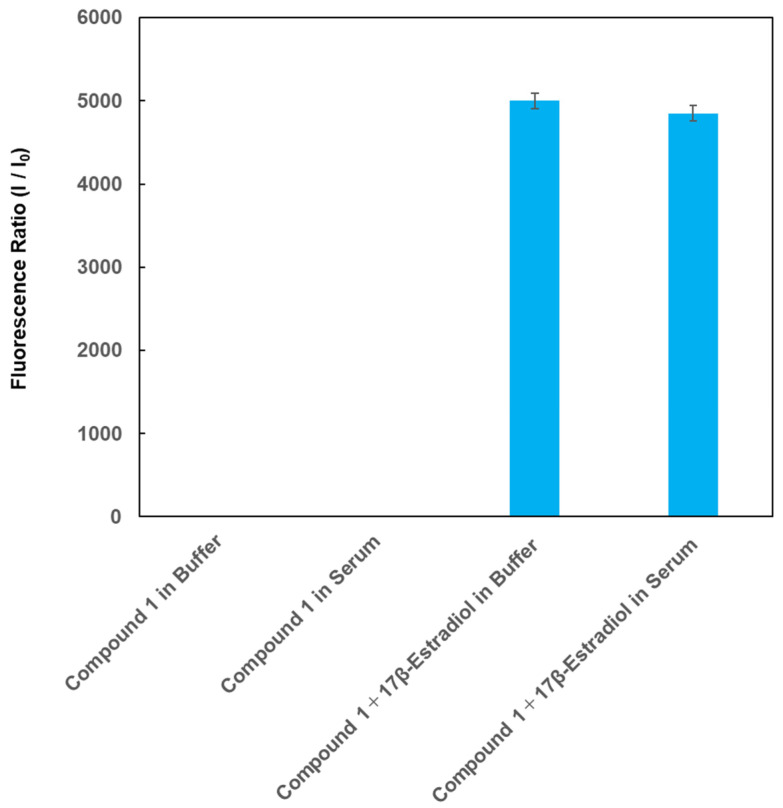
Fluorescence intensity of compound **1** at 569 nm before and after adding 17β-estradiol in control serum or in HEPES buffer solution. [1] = 1.0 nM; [17β-estradiol] = 100 pg/mL; excitation wavelength = 531 ± 20 nm. The y-axis indicates the fluorescence ratio (I/I_0_), where I is the fluorescence intensity of compound **1** at 569 nm before and after adding 17β-estradiol, and I_0_ is the fluorescence intensity of compound **1** at 569 nm.

The selectivity of compound **1** was also evaluated using control serum. Compound **1** and 17β-estradiol were dissolved in control serum or HEPES buffer solution, and the fluorescence intensities of compound **1** were monitored before and after adding 17β-estradiol; the data are shown in Figure 5. The fluorescence intensity of compound **1** itself in control serum was almost the same as that observed in HEPES buffer. A large increase in the fluorescence of compound **1** was observed after adding 17β-estradiol to the control serum and HEPES buffer solution. These findings suggest that compound **1** can selectively detect 17β-estradiol even in the presence of contaminants. This is due to the high selectivity of compound **1** for 17β-estradiol relative to other substances, which may be dependent on the magnitude of complex formation between the nucleic acids and 17β-estradiol.

**Figure 6:**
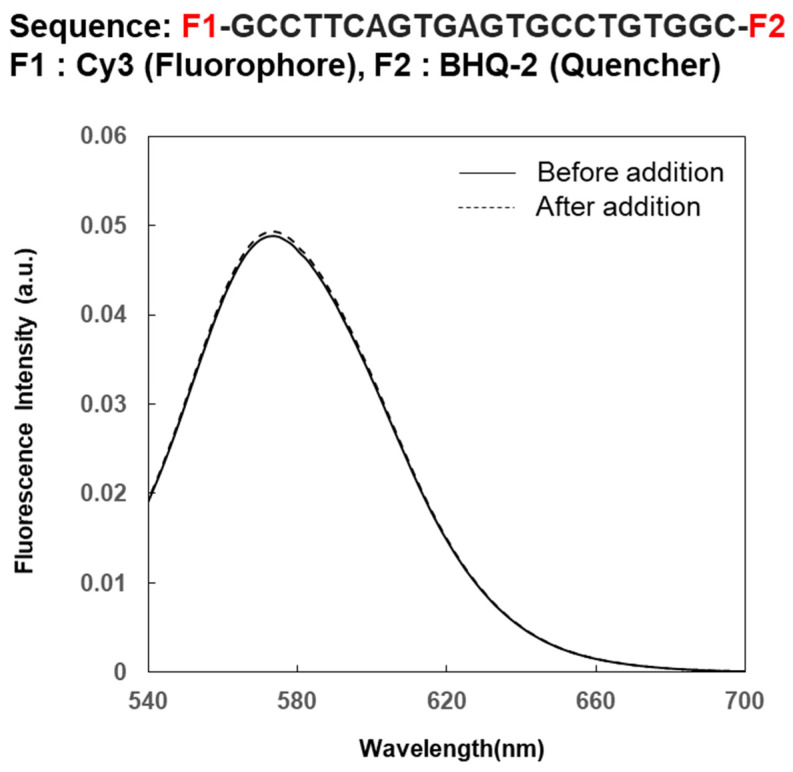
Chemical structure of the reference compound and its sequence (upper), and fluorescence spectral changes in the reference compound before and after adding 17β-estradiol (lower). [reference compound] = 1.0 nM; [17β-estradiol] = 100 pg/mL; excitation wavelength = 530 nm for the fluorescence spectrophotometer; solid line: before adding 17β-estradiol; dotted line: after adding 17β-estradiol.

A reference compound was prepared to confirm whether the nucleic acid sequence in compound **1** contributes to the highly sensitive and selective detection of estradiol. The structure of the reference compound is shown in Figure 6. The number and composition of the nucleic acids in the reference compound are the same as those in the compound, but their sequences were different. The fluorescence intensity of the reference compound when estradiol was added was compared with that of compound **1**. The results are shown in Figure 6. Compound **1** showed a significant increase in fluorescence intensity when estrogen was added, but no change in fluorescence intensity was observed when estrogen was added to the reference compound. These results demonstrate that the nucleic acid sequence in the compound contributes significantly to the highly sensitive and selective detection of estrogen.

**Figure 7:**
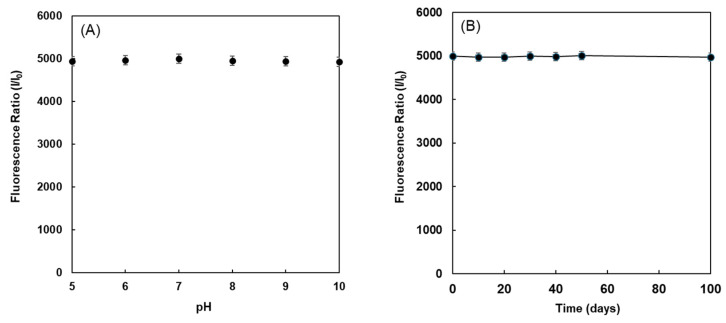
Fluorescence intensities (at 569 nm) of compound **1** after adding 100 pg/mL 17β-estradiol in buffer at different pH (**A**), and storage stability (**B**). [1] = 1.0 nM; [17β-estradiol] = 100 pg/mL; excitation wavelength = 531 ± 20 nm; storage temperature, 4 °C.

This study then examined whether the fluorescence intensity when the compound and estradiol formed a complex was affected by the pH of the solvent. The fluorescence intensities of compound **1** mixed with 100 pg/mL of 17β-estradiol were observed at different pH values ranging from 5 to 10. The data are shown in Figure 7A. Upon binding to 17β-estradiol, fluorescent indicators exhibited high fluorescence intensities, which were nearly unchanged when the pH ranged from 5 to 10. Therefore, this sensing system may satisfy the experimental conditions for the analysis of 17β-estradiol in physiological processes and could be advantageous for real applications.

The stability of fluorescent reagent compound **1** is an important factor for the detection of 17β-estradiol. Compound **1** was stored in a PCR tube at 4 °C, and responses toward 7β-estradiol were observed every 10 days. The fluorescence intensity of the compound after reacting with estrogen remained constant even after the reagent was stored. After six months of storage, the fluorescence intensity was approximately 95% of the initial value as shown in Figure 7B, suggesting that the developed reagent can be stored for a long period of time.

**Figure 8:**
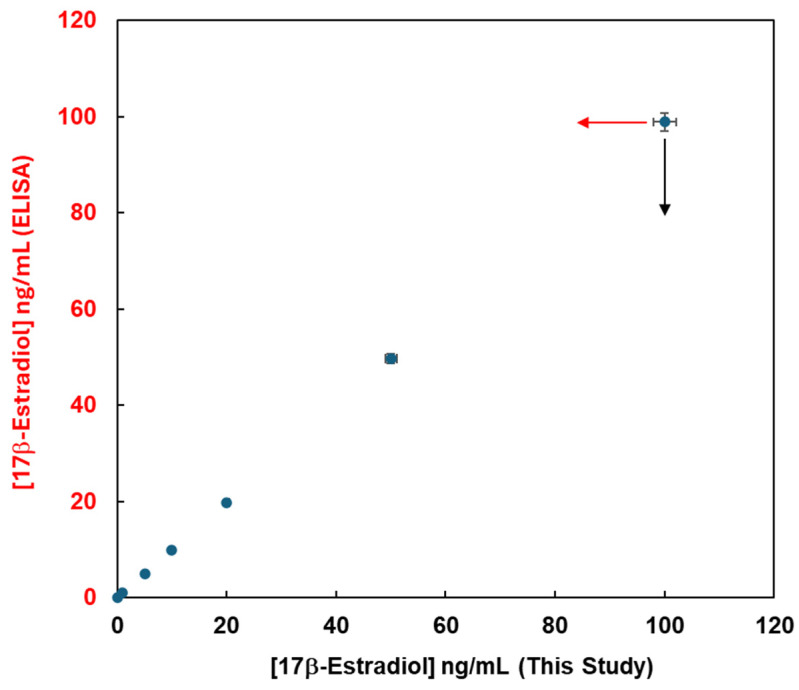
The relationship between 17β-estradiol concentrations obtained using this method on the x-axis and those obtained using ELISA on the y-axis. ELISA: enzyme-linked immunosorbent assay.

To confirm the validity of the measurements obtained in this study, the experimental data in this method were compared with the data obtained using the ELISA method from the viewpoint of the quantitative analysis of 17β-estradiol. Commercially available ELISA kits and the analytical system developed in this study were used for the detection of 17β-estradiol, and the unknown concentrations of 17β-estradiol samples were detected using a calibration curve prepared with known concentrations. Figure 8 shows the 17β-estradiol concentrations obtained with the experimental method on the x-axis and those obtained with ELISA on the y-axis. A good linear relationship was observed between the two different analytical methods, and 17β-estradiol was successfully evaluated. Furthermore, the operation time for this study is about 30 min, whereas that for the ELISA method is about half a day. Therefore, female hormone analysis using the experimental method was more sensitive and easier than that with the conventional ELISA method. The experimental method can be used widely as a high-throughput method for measuring female hormones in human biofluids.

**Figure 9:**
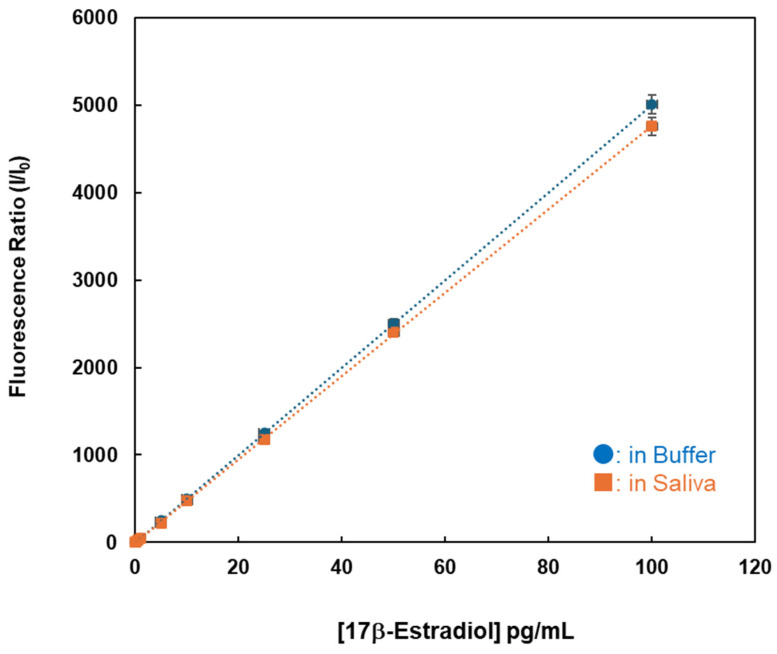
Fluorescence ratio (at 569 nm) of compound **1** after adding various concentrations of 17β-estradiol in buffer and human saliva. [1] = 1.0 nM; excitation wavelength = 531 ± 20 nm; storage temperature, 4 °C.

Finally, estradiol in human saliva was detected using the device developed in this study. After reacting the fluorescent substance with 17β-estradiol dissolved in saliva in a plastic tube, the changes in fluorescence intensities over time were observed. The data are shown in Figure 9. There were linear relationships between the estrogen concentration and the fluorescence intensities, and the fluorescence ratio in saliva was approximately 95% of that in the buffer solution. The 17β-estradiol concentrations (10.0 pg/mL) were quantified in the human saliva with excellent CV values of 2.7%, as shown in Table 1. These findings indicated that the fluorescence indicator formed a stable complex with 17β-estradiol and that interfering substances did not affect the reaction. It is reported that estradiol levels are 9~15 pg/mL in saliva of the female body and 30~400 pg/mL in serum [9]. This sensing system could provide excellent sensitivity and quantification capabilities for the analysis of 17β-estradiol in physiological samples.

Table 2 summarizes representative estradiol detection methods, including immunoassays, chromatographic techniques, and recently reported sensor-based approaches. Key analytical parameters such as limit of detection (LOD), linear range, and assay time are included for comparison. The assay time refers to the intrinsic measurement or reaction time reported in the literature, excluding sample preparation and laboratory workflow. The assay time refers to the intrinsic measurement or reaction time of each method rather than the total clinical workflow duration.

While the liquid chromatography–mass spectrometry (LC/MS) method is highly sensitive and capable of detecting trace amounts of female hormones, it is not a simple analytical method because it requires specialized equipment and skilled techniques.

The ELISA method is applicable to the analysis of a wide range of female hormones, has high quantitative accuracy, and is used not only in laboratories but also in commercial testing facilities such as hospitals and clinics. However, the analytical procedures are complicated, and it takes several hours to a day to obtain the measurement results.

Immunochromatography is a simple analytical method that can be used for self-testing and is characterized by an extremely short detection time of 10–30 min. However, it has the disadvantages of low quantitative accuracy and low sensitivity compared to other analytical methods.

The analysis time required to obtain the results in this study was approximately 30 min, which is roughly the same as that for immunochromatography, and shorter than other fluorescence spectroscopy [26]. Furthermore, since the procedure involves simply mixing the reagent and sample in a tube, it does not require the complicated procedures of LC/MS or ELISA. Furthermore, the method is extremely sensitive compared to ELISA and immunochromatography. This technology could enable the “visualization of hormones,” allowing individuals to monitor their hormone status in real time. Appropriate self-care is expected to be promoted by awareness of real-time hormone fluctuations.

## 4. Conclusions

In this study, a compound with a fluorescent moiety at one end of an aptamer (consisting of a series of nucleic acid bases) and a fluorescence-quenching moiety at the other end was synthesized. This compound was found to be fluorescent on its own but quenched upon binding to female hormones. By mixing this compound with body fluids and measuring the fluorescence quenching, female hormones in body fluids could be analyzed easily. The detection limit was 0.5 pg/mL. The findings from this study could facilitate visualization of daily hormone fluctuations, enabling users to identify signs of PMS, menopausal symptoms, and other conditions. This is expected to aid in proactive self-care, support the prevention of physical discomfort, and promote a sense of security. Furthermore, by objectively monitoring their own bodily rhythms using this analysis system, users may better understand their symptoms, potentially reducing their mental burden.

## Figures and Tables

**Table 1:** Detection of 17β-estradiol using a fluorescent analytical system in human saliva.

Added 17β-Estradiol (pg/mL)	Expected (pg/mL)	Measured (pg/mL) ^a^	CV (%) ^b^	Recovery (%) ^c^
10.00	10.00	9.88	2.7	98.8

^a^ The average value of five independent measurements, ^b^ Coefficient of variation, ^c^ (Measured value) ÷ (Expected value) × 100.

**Table 2:** Comparison of the methods of this study with those of other 17β-estradiol detection studies.

Method	Principle	LOD	Linear Range	Assay Time	Remarks	Reference
ELISA	Antibody-based immunoassay	1–50 pg/mL	1–1000 pg/mL	4 h	High sensitivity; requires multiple washing steps	[11,12]
LC/MS	Chromatography + mass spectrometry	~1–10 fg/mL	Wide (fg/mL–ng/mL)	30–60 min	High accuracy; requires expensive instrumentation	[9]
Immunochromatography	Aggregation of gold nanoparticle	0.1–1 ng/mL	0.5–10 ng/mL	10–30 min	Simple	[25]
Fluorescence (reported)	Aptamer + Fluorophore	0.2 pg/mL	1–100 pg/mL	90 min	Sensitivity varies depending on design	[26]
This work	Aptamer + Fluorophore	0.2 pg/mL	1–1000 pg/mL	30 min	Rapid, sensitive, homogeneous, no sample transfer required	

## Data Availability

The data used in this study are either contained in the article or were randomly generated, as detailed in the article. No external datasets were used in this study.
